# Supplementary material for: Impact of Whole-Genome Sequencing of Mycobacterium tuberculosis on Treatment Outcomes for MDR-TB/XDR-TB: A Systematic Review
Source: Pharmaceutics. 2023 Dec 15;15(12):2782. doi: 10.3390/pharmaceutics15122782 (PMC10747601; doi:10.3390/pharmaceutics15122782)
Supplement: Supplementary file 1 [file pharmaceutics-15-02782-s001.zip › Supplementary File S2.pdf]

Supplementary File S2:

Search Strategy

PubMed	1. ((((((((((((((((((("Multidrug resistant tuberculosis") OR ("tuberculosis, multidrug-resistant"[MeSH Terms])) OR ("MDR TB")) OR ("Multidrug resistant mycobacterium tuberculosis")) OR ("Multidrug resistant tb")) OR ("Multidrug resistant mtb")) OR ("Extensively Drug Resistant Tuberculosis"[MeSH Terms])) OR ("Extensively Drug Resistant Tuberculosis")) OR ("XDR TB")) OR ("Extensively Drug Resistant TB")) OR ("Extensively Drug Resistant mtb")) OR ("Extensively Drug Resistant Mycobacterium tuberculosis")) OR (resistan* tuberculosis)) OR (resistan* mycobacterium tuberculosis)) OR (resistan* tb)) OR (resistan* mtb)) OR (drug resistan* tuberculosis)) OR (drug resistan* tb)) OR (drug resistan* mtb)) OR (drug resistan* mycobacterium tuberculosis)<br><br>2. ((((((((((((((((("treatment outcome"[MeSH Terms]) OR ("treatment outcome")) OR (clinical outcome*)) OR ("Clinical application")) OR (treatment outcome*)) OR ("patient outcome assessment"[MeSH Terms])) OR ("patient outcome assessment")) OR (patient* outcome*)) OR ("outcome assessment")) OR ("treatment response")) OR ("long-term outcome")) OR ("treatment failure"[MeSH Terms])) OR ("treatment failure")) OR (prognosis[MeSH Terms])) OR (Prediction outcome*)) OR (Therapeutic* outcome*)) OR ("cohort study")) OR ("clinical trial")<br><br>3. (((((((((((("whole genome sequencing"[MeSH Terms]) OR ("whole genome sequencing")) OR (WGS)) OR ("high-throughput nucleotide sequencing"[MeSH

	Terms])) OR ("high-throughput nucleotide sequencing")) OR ("next-generation sequencing")) OR (NGS)) OR ("complete genome sequencing")) OR (mutation*)) OR (gene* mutation*)) OR ("genomic mutation")) OR (gene sequenc*)) OR (genomic sequenc*)<br>4. 1 AND 2<br>5. 4 AND 3 Filters: Multicenter Study, Observational Study, Randomized Controlled Trial, Humans, English, from 2000/1/1 - 2020/12/31
